# The Treatment of Inflammation of the Eustachian Tube

**Published:** 1882-06

**Authors:** 


### THE TREATMENT OF INFLAMMATION OF THE EUSTACHIAN TUBE.

In a recent lecture, Dr. George Strawbridge, of Philadelphia (*Virginia Medical Monthly*, March, 1882), gives some excellent practical directions for the treatment of inflammation of the Eustachian tube, an affection which is usually but little understood by the general practitioner. The indications in the treatment are: first, the arrest of the inflammation; second, the management of the Eustachian tube; third, bringing the drum-membrane back into its proper position; and fourth, to dispose of the products of inflammation.

First, arrest of inflammation: If there is a feeling of fulness and pain in the ear, and signs of hyperæmia are present, apply a leech every one, two, or three days for a week or ten days. A blister behind the ear is sometimes useful. Tincture of iodine on the mastoid often does well. Painting the drum membrane with a solution of silver nitrate. (He has used an eighty-grain solution with good results.) The ear should be protected against cold. The second indication is met by spraying the throat, the upper pharyngeal space and the nares if necessary, with a solution of silver nitrate (5–80 grains to the ounce), or carbolic acid (1–2 drams to the pint of water). The remedy will be a little more effective if applied directly against the mouth of the tube. The Eustachian catheter or Strawbridge's faucial catheter may be used. The solution must not be used too frequently, and care must be taken not to throw it into the tympanum. A 20–80 grain solution of silver nitrate is quite proper to use.

To restore the drum membrane to its proper position. and to open the tube, inflation is indicated. The simplest means of doing this is to use a flexible tube, one end to be placed in the external meatus and the other in the patient's mouth.

By slight suction, the drum membrane will resume its proper position. There are some objections to the method, and it is not very efficient. If there is complete obstruction of the Eustachian tube, of course the method will not answer. Paracentesis of the drum membrane is often serviceable for the relief of the membrane. It more effectually relieves tinnitus than any other remedy.

To dispose of the products of inflammation, mercury and iodide of potassium are about the only remedies needed. The former can be efficiently used by inunction for four to eight days, followed by ten days or two weeks of the iodide; then return to mercury if necessary. Mixed treatment may be used. The patient's general condition must be taken account of. Good food, clothing, fresh air, and cleanliness are essential.

